# Lessons Learned From an Effectiveness Evaluation of Inlife, a Web-Based Social Support Intervention for Caregivers of People With Dementia: Randomized Controlled Trial

**DOI:** 10.2196/38656

**Published:** 2022-12-07

**Authors:** Hannah Liane Christie, Alieske Elisabeth Henrike Dam, Martin van Boxtel, Sebastian Köhler, Frans Verhey, Marjolein Elisabeth de Vugt

**Affiliations:** 1 Department of Psychiatry and Neuropsychology School for Mental Health and Neuroscience Maastricht University Maastricht Netherlands

**Keywords:** dementia, Alzheimer's, neurodegenerative, caregiver, caregiving, digital health, eHealth, mHealth, Information communication technology, RCT, randomized controlled trial, social support, support platform, online platform, web-based, internet-based, peer-support, informal support, social interaction, support network

## Abstract

**Background:**

Informal care for people with dementia not only affects the well-being of the primary caregiver but also changes their roles and interactions with the social environment. New online interventions might facilitate access to social support. Recently, an online social support platform, Inlife, was developed in the Netherlands and aims to enhance social support and positive interactions in informal support networks.

**Objective:**

This study aimed to evaluate the effectiveness of Inlife for caregivers of people with dementia.

**Methods:**

A randomized controlled trial with 96 caregivers of people with dementia was performed. Participants were randomly assigned to the Inlife intervention or the waiting list control group. After 16 weeks of Inlife use, the waiting list control group could start using Inlife. Effects were evaluated at baseline (T_0_), 8 weeks (T_1_), and 16 weeks (T_2_). The 16-week follow-up assessment (T_2_) served as the primary endpoint to evaluate the results for the primary and secondary outcome variables evaluated with online self-report questionnaires. The primary outcomes included feelings of caregiver competence and perceived social support. The secondary outcomes included received support, feelings of loneliness, psychological complaints (eg, anxiety, stress), and quality of life.

**Results:**

No significant improvements were demonstrated for the intervention group (n=48) relative to the control group (n=48) for the primary outcomes (feeling of carer competence: b=–0.057, 95% CI –0.715 to 0.602, *P*=.87; perceived social support: b=–15.877, 95% CI –78.284 to 46.530, *P*=.62) or any secondary outcome. This contrasts with our qualitative findings showing the potential of Inlife to facilitate the care process in daily life. Adherence was not optimal for all Inlife users. Additional per-protocol and sensitivity analyses also revealed no beneficial results for high active Inlife users or specific subgroups. Inlife users were more active when part of a larger network.

**Conclusions:**

Researchers should be modest regarding the effectiveness of online caregiver interventions in terms of quantitative measures of well-being and quality of life. Nevertheless, online tools have the potential to facilitate the caregiver process in daily life. Lessons learned include the importance of harnessing the power of human interaction in eHealth, making use of the user’s social capital, and the need to develop research methods that can identify benefits in daily life that are ecologically valid for caregivers.

**Trial Registration:**

Netherlands Trial Register NTR6131; https://trialsearch.who.int/Trial2.aspx?TrialID=NTR6131

**International Registered Report Identifier (IRRID):**

RR2-10.1186/s13063-017-2097-y

## Introduction

### Dementia and Caregiving

A substantial part of the care for people with dementia is provided by informal caregivers such as family, friends, and relatives [1]. Consequently, informal caring not only affects the well-being of the primary caregiver but also changes their roles and interactions with the social environment [2,3]. Although some involved caregivers report the benefits of caring, such as the strengthening and enrichment of mutual relationships, family cohesion, or personal growth [4], others face negative consequences of caring on their physical and mental health or experience increased burden due to the growing dependence of the people with dementia on their environment [2,5]. During the disease process, caregivers of people with dementia are at risk of becoming socially isolated since they might become homebound due to decreased mobility, memory problems, behavioral problems, denial of the disease, or experienced stigma [6-8].

To prevent social isolation and loneliness in caregivers and people with dementia, support is needed after a diagnosis has been made. Previous qualitative studies with carers of people with dementia have shown that there is a postdiagnostic care mismatch between the supply and demand of informal support. The authors advocated introducing early access to tools to improve open communication and facilitate positive social engagement in dementia care networks before carers might become overburdened and while they still have the resources to learn new caregiving skills [9,10]. Existing psychosocial interventions focus on psychoeducation, skill building, and psychotherapeutic counselling based on techniques derived from cognitive behavioral therapy [11-13]. It has been demonstrated that individually tailored, multicomponent interventions offered to both the caregiver and the people with dementia have positive effects on burden, anxiety, and depression [11-13]. However, the effects are generally small to moderate, and available studies are limited in their methodological quality [14].

### Online Interventions to Support Caregivers

With the introduction of the Internet and social media in daily life in recent decades, online interventions have become a new avenue for caregiver support. Recent online intervention studies that contained multiple individual tailored elements of psychoeducation, peer support, skills training, and health assessment have demonstrated improved caregiver well-being, including conﬁdence, self-efﬁcacy, and lower levels of depression [15-17]. Therefore, online caregiver support might be an alternative to traditional counselling and support interventions for several reasons. Online tools are always available, regardless of distance, time, and mobility constraints, and provide easily accessible, low-cost support and caregiver empowerment [18]. Using online tools might elicit support-seeking behavior and engagement in social activities to cope better with stressful situations [19] or enhance feelings of competence [20,21] to deal with the challenges faced in the caregiving process. Additionally, through accessibility and widespread reach, online interventions can lower the threshold to involve the caregiver’s social network and either seek or provide social support by increasing openness and positive interaction [9]. This might prevent social isolation and increase feelings of competence in caregivers. Although results from online network interventions are promising [22], the use in informal caregiver social networks is relatively new and not yet studied.

### Inlife

Therefore, the online social support intervention “Inlife” was developed for caregivers of people with dementia and made available in the Netherlands [23]. Inlife intends to help caregivers of people with dementia overcome barriers to seeking help while also removing barriers for loved ones and other individuals involved in the dementia care network to offer help. Using the “Inlife platform,” the primary caregiver is encouraged to invite family, friends, and significant others into their personalized support circles. The functionalities in the intervention include Profile (personal information), Circles (layers of caregivers with different privileges), Helping (general overview to place and receive responses to help requests), Timeline, Calendar, Personal Messages, Care Book, and Compass (information about dementia-related topics). Details of these functionalities are described elsewhere [24]. Inlife is currently being called “Myinlife” after being adopted by a societal partner, the Dutch Alzheimer’s association (Alzheimer Nederland).

### Aim

The aim was to evaluate the effectiveness of Inlife in a randomized controlled trial (RCT) over a 16-week period [24]. Primary outcomes were feelings of competence and perceived social support. Secondary outcomes were received support, feelings of loneliness, psychological complaints, and quality of life. We hypothesized that, compared with care as usual, use of the Inlife intervention would lead to change in both the primary and secondary outcome measures.

## Methods

### Participants and Design

Between June 2016 and June 2017, informal primary caregivers of people with dementia of all subtypes and stages were recruited via online advertisement, newsletters, and social media channels of the Dutch Alzheimer Association; regional dementia community services; and memory clinics or other relevant care institutions. The inclusion criteria were (1) being a primary, informal caregiver of a person diagnosed with dementia of any subtype; (2) having Internet access; and (3) having basic (tablet) computer knowledge as assessed by the researcher. Participants were excluded if they were unavailable for longer than 4 weeks during the study period or had serious health problems incompatible with participation as assessed by the study staff.

After the baseline assessment, participants were randomly assigned to either the intervention or a waiting list control group. The intervention group participated in the Inlife intervention. The waiting list control group received care as usual and was able to start with Inlife after 16 weeks.

Randomization was performed using a computerized sequence generator for block randomization with variable sizes of 4, 6, and 8 (for details, see [24]). The follow-up assessments were completed online using a secure, custom-designed query system.

### Ethical Approval

The study was approved by Ethical Committee of the Faculty of Psychology & Neuroscience of Maastricht University (ERCPN-172_20_03_2016_A1; Dutch Trial Register trial number: NTR6131) More detailed information about the study design is presented elsewhere [24]. The CONSORT eHEALTH checklist is presented as Multimedia Appendix 1.

### Procedure

Participants were screened by telephone to check eligibility. Subsequently, the participating caregivers provided online informed consent. Assessments were collected online at 3 time points: pre-intervention (T_0_), 8 weeks (T_1_), and 16 weeks (T_2_). The 16-week follow-up assessment served as a primary endpoint to compare group effects [24]. Because 2 weeks was deemed an adequate amount of time to become familiar with the platform, 2 weeks after registration on the Inlife platform, participants were contacted by phone to reflect on user experiences. This was done to facilitate engagement, stave off attrition, and resolve any initial queries about the platform.

### Conditions

The intervention group had access to Inlife, an online social support platform for informal caregivers and people with dementia aimed at strengthening positive interactions and social support. All users had a secure username and password combination to access the website and complementary app for smartphones and tablets. Participants could use Inlife in a flexible manner and at their own pace. The platform remained accessible to them after the 16-week study period. Participants in the control group remained on the waiting list for 16 weeks and received care as usual. Care as usual entailed that the participants continued with any routine care they may receive, such as consultations with their general practitioner or dementia case manager. After the 16-week follow-up assessment (T_2_), they had the opportunity to register on the Inlife platform.

### Measures

A short overview of applied self-reported measurement instruments is provided in this section. More details can be found elsewhere [24].

#### Primary Outcomes

First, caregiver sense of competence was assessed using the Short Sense of Competence Questionnaire (SSCQ), which consists of 7 items that refer to caregivers’ feelings of being capable of caring for the people with dementia. The total score ranges from 0 to 7. The SSCQ has been evaluated as a valid and reliable instrument in caregiver research [25]. Next, perceived support was measured using the 12-item Multidimensional Scale of Perceived Social Support (MSPSS). The total score ranges from 12 to 84. The psychometric characteristics of the scores were sufficient in clinical [26] and nonclinical populations [27].

#### Secondary Outcomes

Received support was measured using the 12-item Social Support List-Interactions (SSL12-I); the total score ranges from 12 to 48. Good internal reliability has been previously demonstrated [28]. Feelings of loneliness were measured using the 11-item Loneliness Scale (LS), with total scores ranging from 11 to 33. The psychometric properties were sufficient [29]. The 6-item Lubben Social Network Scale (LSNS-6) was used to assess the number of friends and family ties. The total score ranges from 0 to 30. The LSNS-6 has been validated in an older sample [30]. Anxiety and depression symptoms were assessed using the Hospital Anxiety and Depression Scale (HADS). The total score ranges from 0 to 42, and the Dutch version of the HADS has demonstrated good reliability and validity [31]. Perceived stress was measured with the 10-item Perceived Stress Scale (PSS), with a total score ranging from 0 to 40. Sufficient psychometric quality has been demonstrated [32]. Perseverance time for the caregiver was measured by a single item, with scores ranging from 1 to 4. The scale was specifically validated for informal caregivers of people with dementia [33]. Domains of quality of life or capability were measured using the Investigating Choice Experiments for the Preferences of Older People Capability Measure for Older People (ICECAP-O). The summary score ranges from 0 (no capability) to 1 (full capability), and the scale has been sufficiently validated [34]. The impact of caring on quality of life was assessed using the Care-Related Quality of Life Scale (CarerQol). Scores range between 7 and 21. The psychometric properties were sufficient [35,36]. Furthermore, at baseline, (socio-)demographics of the caregivers and care recipients were collected including age, sex, education, and hours of contact with and hours caring for the people with dementia. Additionally, the number of clicks on the Inlife website was collected to measure actual usage of the platform (results reported elsewhere) [24].

### Sample Size Calculation

The sample size calculation was based on previous intervention studies of caregivers of people with dementia with the SSCQ as an outcome measure [16,35], using differences between intervention and control groups at follow-up with an assumed mean effect size of Cohen d of 0.5 (medium effect). With an alpha of .05 and power of 80%, we aimed to include 102 primary caregivers (51 participants per group). Allowing for a 20% loss to follow-up, we aimed to enroll a total of 122 caregivers into the study.

### Statistical Analysis

Statistical analyses were conducted in SPSS Version 24.0 (IBM Corp, Armonk, NY). Before analysis, data were examined for missing values, outliers, and normality. Potential differences between the intervention and control groups in baseline characteristics and outcome variables at baseline and the 16-week follow-up, which might require adjustment for such differences, were tested using either *t* tests for continuous variables or Chi-square tests for categorical variables. Since there were missing values, we compared the baseline characteristics of study completers and the participants with missing values. A separate analysis revealed that missingness was related to the sex of the person with dementia. Since missing values were not completely at random, data were analyzed according to intention-to-treat (ITT) principles applying a multiple imputation strategy. We used the Markov chain Monte Carlo method in SPSS to produce 10 data sets. These were subsequently analyzed, and estimates were pooled using the Rubin rule [36]. Subsequently, we performed a per-protocol (PPT) analysis including only the caregivers in the analyses that used Inlife until 16 weeks in the intervention group. A subsequent sensitivity analysis was conducted by contrasting the high active versus the low active Inlife users with the control group. The intervention group was split into high active and low active Inlife users based on the median total number of clicks on the platform [24].

To test the differences in the outcome variables in the intervention group and waiting list control group, we performed linear regression analysis on the imputed data sets, with outcomes from the T_2_ assessment as dependent variables (ie, the primary endpoint at 16 weeks; after this period, the waiting list control group was able to start using Inlife). The primary and secondary outcome variables at the T_2_ follow-up were included in the model as dependent variables, and group was included as the between-subjects variable. Statistically significant baseline differences between the treatment arms (eg, age of the person with dementia) were included as covariates. Each outcome measure was assessed as a dependent variable in separate analyses. For a variable that was positively skewed, a cubic transformation was applied to better approximate a normal distribution. Subsequently, to test the changes in the primary outcome measures over time, data were analyzed performing a linear mixed model (LMM) on the nonimputed data set. This analysis estimates the fixed effects of the regression slopes, indicating the changes during the intervals (T_0_-T_1_ and T_1_-T_2_) in the intervention and waiting list control groups. This procedure allows for modelling the rate of change in the primary outcome variables for the caregivers who did not receive the intervention compared with the caregivers who received the intervention (T_0_-T_2_). This analysis accounts for within-subject correlations between repeated measures using random (ie, individual-specific) effects, thus accounting for the hierarchical structure in the data (ie, time nested in individuals). Additionally, LMM handles missing values efficiently under the missing at random assumption if variables that are associated with missingness are included in the analyses using maximum likelihood estimates for the missing observations. Hence, it is suitable for ITT analysis [37]. Random effects for the intercept only were specified because likelihood ratio testing revealed that this model fit the data better than adding a random slope or adjusting for correlated residuals. To model the effect of the intervention on the primary outcome variables over time, we entered a group-by-time interaction term as a dummy variable for each of the follow-ups to allow for nonlinear effects. The model was adjusted for baseline differences (eg, age of the person with dementia) and associations with missingness (eg, sex of the person with dementia). All tests were 2-tailed with an alpha level of .05.

## Results

### Participants and Descriptive Statistics

**Figure 1:**
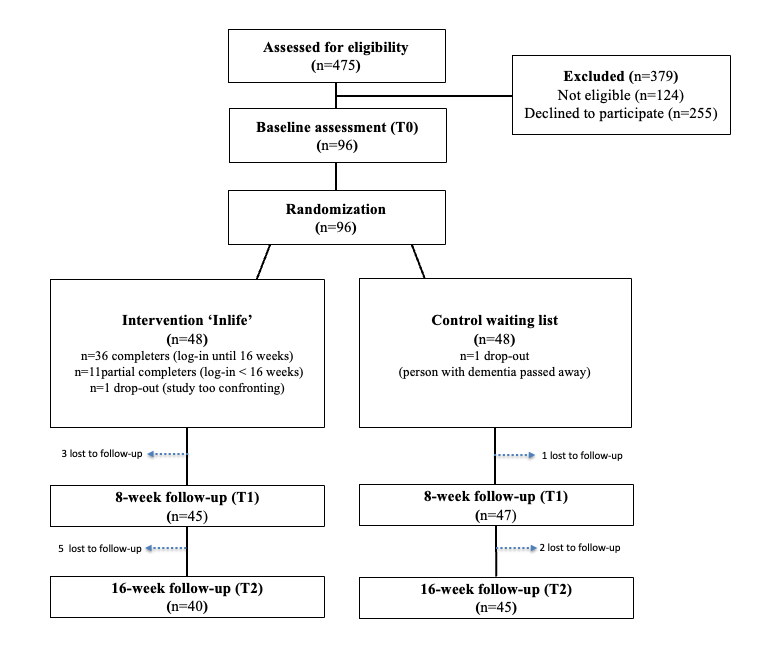
Participant flow diagram according to Consolidated Standards of Reporting Trials (CONSORT).

A total of 475 caregivers were approached to participate in the study. In total, 379 people were excluded: 124 people were excluded due to ineligibility, and 255 people were excluded due to the fact that they declined to participate. Subsequently, 96 informal caregivers who signed informed consent were included. In total, 96 caregivers signed informed consent and were randomly assigned to either the Inlife intervention group (n=48) or the waiting list control group (n=48). Reasons for declining participation are described elsewhere [24]. Of the 96 randomized participants, 89 completed the 16-week follow up (T_2_). Figure 1 depicts the flowchart of study participation. The baseline characteristics for completers and noncompleters did not differ significantly. The baseline characteristics of the participants are presented in Table 1. For the majority of variables, there were no significant differences between the groups, except for age of the person with dementia (*t*_94_=–2.05, *P*=.04) and baseline scores on the SSCQ (*t*_93_=–2.65, *P*=.01) and ICECAP-O (*t*_93_=–2.81, *P*=.006), which were significantly different. Therefore, these variables were included as covariates in the analysis. The high active Inlife users had a larger number of circle members in their network (mean 9.4, SD 5.2) compared with the low active users (mean 3.3, SD 3.7).

**Table 1:** Characteristics of the intervention and control groups.

Variables				Intervention (n=48)		Control (n=48)		*P* value
**Baseline**								
	Caregiver age (years), mean (SD) range)		58.1 (11.8)		55.7 (13.6)		.35	
	Caregiver age (years), range		26-84		22-82		N/A^a^	
	**Sex, n (%)**							.51
		Male	17 (35.4)		14 (29.2)			
		Female	31 (64.6)		34 (70.8)			
	Caregiver education (years), mean (SD)		13.1 (5.1)		14.1 (5.7)		.36	
	Length of caregiving (years), mean (SD)		6.2 (7.8)		4.2 (4.6)		.14	
	Weekly caregiving (hours), mean (SD)		33.9 (44.9)		27.9 (48.0)		.53	
	**Caregiver relationship (n, %)**							.30
		Spouse/partner	21 (43.8)		13 (27.1)			
		Son or daughter (-in law)	24 (50.0)		33 (68.7)			
		Brother or sister	1 (2.1)		1 (2.1)			
		Other	2 (4.2)		1 (2.1)			
	Number of other involved caregivers, mean (SD)		2.2 (2.2)		2.5 (2.3)		.59	
	Care recipient age (years), mean (SD)		75.0 (10.8)		79.1 (8.8)		.04	
	Care recipient age (years), range		47-91		55-92		N/A	
	Care recipient education (years), mean (SD)		10.2 (4.9)		11.1 (6.5)		.43	
	**Type of dementia (n, %)**							.47
		Alzheimer’s disease	25 (52.1)		19 (39.6)			
		Frontotemporal dementia	5 (10.5)		2 (4.2)			
		Vascular dementia	6 (12.5)		9 (18.6)			
		Dementia with Lewy bodies	1 (2.1)		3 (6.3)			
		Mixed dementia	2 (4.2)		5 (10.5)			
		Dementia not otherwise specified	9 (18.6)		10 (20.8)			
	**Living situation (n, %)**							.13
		Home	41 (85.4)		35 (72.9)			
		Nursing home	7 (14.6)		13 (27.1)			
**Outcome variables at baseline and the 16-week follow-up**								
	**SSCQ^b^, mean (SD)**							
		Baseline (n=95)	3.8 (1.9)		4.7 (1.6)		.01	
		16-week follow-up (n=89)	3.7 (1.9)		4.5 (1.7)		.05	
	**MSPSS^c^, mean (SD)**							
		Baseline (n=96)	63.3 (13.0)		68.3 (11.7)		.055	
		16-week follow-up (n=89)	63.8 (16.5)		66.5 (12.8)		.38	
	**SSL-12^d^, mean (SD)**							
		Baseline (n=96)	29.8 (7.1)		31.8 (8.3)		.20	
		16-week follow-up (n=90)	29.6 (7.6)		32.6 (7.5)		.06	
	**LS^e^, mean (SD)**							
		Baseline (n=96)	3.9 (3.4)		3.6 (3.5)		.64	
		16-week follow-up (n=89)	4.4 (3.8)		3.5 (3.9)		.28	
	**LSNS-6^f^, mean (SD)**							
		Baseline (n=95)	17.0 (6.6)		18.4 (4.9)		.26	
		16-week follow-up (n=89)	17.7 (6.6)		18.4 (6.1)		.33	
	**PSS^g^, mean (SD)**							
		Baseline (n=95)	14.2 (6.6)		13.7 (6.6)		.70	
		16-week follow-up (n=89)	15.5 (6.9)		14.1 (8.0)		.40	
	**HADS^h^, mean (SD)**							
		Baseline (n=95)	22.8 (2.2)		22.0 (2.9)		.15	
		16-week follow-up (n=89)	22.4 (2.6)		22.9 (2.5)		.37	
	**PT^i^, mean (SD)**							
		Baseline (n=95)	5.4 (0.9)		5.2 (1.2)		.42	
		16-week follow-up (n=89)	4.6 (1.2)		4.5 (1.5)		.84	
	**CarerQol^j^, mean (SD)**							
		Baseline (n=95)	5.6 (1.8)		5.9 (2.1)		.47	
		16-week follow-up (n=89)	6.0 (2.0)		6.0 (2.0)		.99	
	**ICECAP-O^k^, mean (SD)**							
		Baseline (n=95)	0.78 (0.13)		0.84 (0.10)		.006	
		16-week follow-up (n=89)	0.77 (0.14)		0.83 (0.12)		.03	

^a^N/A: not applicable.

^b^SSCQ: Short Sense of Competence Questionnaire.

^c^MSPSS Multidimensional Scale of Perceived Social Support.

^d^SSL-12: Social Support List 12-Interactions.

^e^LS: Loneliness Scale.

^f^LSNS-6: Lubben Social Network Scale.

^g^PSS: Perceived Stress Scale.

^h^HADS: Hospital Anxiety and Depression Scale.

^i^PT: perseverance time.

^j^CarerQol: Care and Quality of Life scale.

^k^ICECAP-O: Investigating Choice Experiments for the Preferences of Older People Capability measure for Older People.

### Intervention Effects

Primary and secondary outcome measures were compared between groups (intervention vs waiting list control) after 16 weeks (T_2_). Table 2 shows the results of the ITT analysis. Overall, no significant effects in favor of the intervention group compared with the control group were found. The PPT analysis also did not yield any significant effects. Considering our heterogeneous group, we performed a post hoc sensitivity analysis splitting the data into low active and high active users (Multimedia Appendix 2), caregiver relationship status (spouse vs children or others at distance), and living situation of the person with dementia (home vs institution). This analysis revealed no significant differences between the caregiver groups at T_2_ (see Multimedia Appendix 2).

For the primary outcome variables, we explored the rate of change over time, as shown in Table 3. For caregiver feelings of competence (SSCQ), the analysis demonstrated a significant group difference at baseline, but no significant overall interaction between group and time was found, indicating that change over time in caregiver feelings of competence was not explained by the intervention (see Multimedia Appendix 3). Similarly, the caregiver perceived support (MPSSS) analysis revealed no overall interaction between group and time, indicating that change over time in caregiver-perceived social support was not explained by the intervention (see Multimedia Appendix 4).

**Table 2:** Intention-to-treat (ITT) analyses comparing the intervention group (n=48) and control group (n=48) at the 16-week follow-up, showing the pooled statistics of the linear regression analyses of the 16-week follow-up outcome measures adjusted for the age of the person with dementia.

ITT^a^	B	SE	Group *P* value	95% CI
SSCQ^b,c^	–0.057	0.335	.87	–0.715 to 0.602
MSPSS^d,e^	–15.877	31.841	.62	–78.284 to 46.530
SSL-12^f^	–2.511	1.551	.11	–5.550 to 0.528
LS^g,h^	0.576	0.792	.47	–0.976 to 2.128
LSNS-6^i^	–1.586	1.495	.29	–4.524 to 1.352
PSS^j^	0.893	2.074	.67	–3.196 to 4.982
HADS^k^	–0.541	0.536	.31	–1.591 to 0.509
PT^g,l^	0.031	0.524	.95	–1.011 to 1.073
CarerQol^m^	0.097	1.022	.93	–1.928 to 2.122
ICECAP-O^d,n^	–0.013	0.024	.59	–0.060 to 0.034

^a^ ITT is based on a multiple imputation Markov chain Monte Carlo method with 10 iterations.

^b^Adjusted for baseline scores.

^c^SSCQ: Short Sense of Competence Questionnaire.

^d^This variable was negatively skewed, and a cubic transformation was used.

^e^MSPSS: Multidimensional Scale of Perceived Support.

^f^SSL-12: Social Support List 12-Interactions.

^g^This variable was skewed, but no transformation could better approach a normal distribution; therefore, raw data are presented.

^h^LS: Loneliness Scale.

^i^LSNS-6: Lubben Social Network Scale.

^j^PSS: Perceived Stress Scale.

^k^HADS: Hospital Anxiety and Depression Scale.

^l^PT: perseverance time.

^m^CarerQol: Care and Quality of Life scale.

^n^ICECAP-O: Investigating Choice Experiments for the Preferences of Older People Capability measure for Older People.

**Table 3:** Differences in rates of change over time for the primary outcome measures between the intervention and control groups (n=96), assessed using a linear mixed model (group, time, group x time, age of person with dementia, sex of person with dementia).

Parameter			Baseline				8-week follow-up				16-week follow-up					*F* statistic for group x time (df)^a^
			B		95% CI		B		95% CI		B		95% CI			
**SSCQ^b^**																
	Group^c^	–0.96		–1.65 to –0.26		0.32		–0.24 to 0.88		0.20		–0.37 to 0.77		0.66 (2,183)		
**MSPSS^d^**																
	Group^c^	–4.6		–10.35 to 1.07		–0.17		–4.39 to 4.05		1.17		–3.11 to 5.44		0.22 (2,181)		

^a^Test of overall interaction between group (intervention, control) and time (baseline, 8-week follow-up, 16-week follow-up).

^b^SSCQ: Short Sense of Competence Questionnaire.

^c^The control group is the reference group.

^d^MSPSS: Multidimensional Scale of Perceived Social Support.

## Discussion

### Main Findings

This RCT evaluated Inlife, an online social support intervention for caregivers of people with dementia. No significant improvements in the primary or secondary outcome variables were demonstrated for the intervention group relative to the control group. Additional PPT and sensitivity analyses revealed no beneficial results for the high active Inlife users or specific subgroups of caregivers (ie, spouse vs children or community dwelling vs institutionalized). However, the results indicated that users in general were more active when they had a larger number of people in their Inlife network. Furthermore, active users tended to have slightly longer care duration.

### Lessons Learned

#### Care Circle Size Is Linked to Inlife Engagement

A first important lesson learned was how Inlife care circle size is linked to engagement with the platform. High active users tended to have a larger number of circle members on Inlife. Previous research also showed more beneficial results of an online support intervention for caregivers with larger informal social networks [37]. Similarly, Inlife might be especially helpful for individuals with an already large social network facilitating openness, involvement, and information flow in their daily life.

Conversely, it is possible that the Inlife intervention could have unintentionally induced a heightened awareness of one’s lack of available support in circles with a low level of responsiveness, which may otherwise not have been salient. This could be a contributing factor to the suboptimal compliance to Inlife. This finding is in line with our qualitative results described elsewhere [38]. Compliance issues are not uncommon in eHealth intervention studies and are likely to lessen their effectiveness [39].

#### Harnessing Social Capital and Embedding Inlife in the Care Context

It is important to note that the observed circle size across Inlife users is probably not representative of the actually available social capital. Indeed, the number of people in the social networks of the low and high active Inlife users was not significantly different at baseline, as measured by the LSNS-6 (see Multimedia Appendix 5). This indicates that some caregivers might still experience difficulties in recruiting people in their social network to join the Inlife platform. This hinders the full use of Inlife, and it raises the question of how we could help Inlife users to involve and expand their care circles and social capital. Additional offline guidance and information could help to extend access to available social capital and to overcome the existing threshold, stigma, and barriers to seeking support [9,23]. Health care professionals could help increase awareness of caregivers’ social support needs and already existing social capital, potentially also by making the link to local peer support services and offline networks, such as Alzheimer Cafés. This also provides the opportunity to connect online support to offline support, where potential Inlife users could be introduced to both health care professionals and peers who might provide upfront support as well as aid with actual Inlife usage to increase compliance rates and alleviate potential negative effects of low care circle responsiveness. Previous research has demonstrated that guidance by a coach could be a valuable contribution to online interventions, as blended eHealth interventions (that is, eHealth interventions that combine online and offline support elements) for caregivers appear to be more effective than nonblended interventions [15]. Moreover, integrating eHealth interventions for caregivers of people with dementia into existing (dementia) care organizations is an important determinant of their sustained implementation success [40]. This approach would necessitate thorough training and monitoring of the implementing health care professionals, as research has shown that implementer self-efficacy and sense of ownership are important predictors of sustainable implementation of online interventions for caregivers of people with dementia [41].

#### Considering Innovative Research Designs

Recently, researchers have been more critical of the gold standard of the RCT design for the evaluation of eHealth interventions [42]. Although the RCT is an established and proven method to gain insight into eHealth effectiveness and mechanisms, they are time and resource-intensive and often result in a lack of important, qualitative implementation data [43]. Currently, staying up to date with technological advancements is a challenge due to the expansive time frame of typical effectiveness studies. One way of developing eHealth interventions that are suitable to implementation when proven effective is by using more flexible research designs [44]. Inspiration for methods to evaluate (new functionalities to) eHealth interventions can be gained from industry, where many commercial platforms use real-time evaluations to gain feedback from users. These can include pop-ups, which ask the user to rate their experiences, or the launch of different versions of the same functionality in order to assess which of the 2 versions is more successful [40]. It is possible that the retrospective measurements used in this study could not capture the kinds of practical benefits (ie, increased time savings and positive interactions) that are highly important and ecologically valid for informal caregivers. Previous research has demonstrated that experience sampling methodology can provide both caregivers and clinicians with more detailed, ecologically valid information about caregiver well-being in real time [21]. This is a promising measurement method for the evaluation of online tools such as Inlife, as they are applied during daily life and show a more complete, variable picture, rather than a retrospective summary.

### Strengths and Limitations

A first important strength of Inlife includes its development through co-design with potential users and its feasibility testing in a pilot evaluation, as recommended by the Medical Research Council framework [45]. This improved the usability and face validity of the platform. Second, the effects of Inlife were evaluated with a robust research design and statistical approach via an RCT that applied both ITT analysis and PPT analysis.

However, this study also has several limitations (in addition to the issues relating to the study's RCT design and retrospective measurements discussed in the section “Considering Innovative Research Designs”). First, the waiting list control design of the study might have affected group differences, as the waiting list control group could have had a longer anticipatory experience than the intervention group. However, eventually, the design enabled all interested caregivers to gain access to the Inlife platform. Second, this study’s sample was heterogeneous in nature, consisting of both spousal caregivers and children of people with dementia living either in the community or in care institutions. We selected this broad population considering the exploratory nature of our study to increase the generalizability of our findings to the general population. However, the power of the present study was insufficient to reveal the effectiveness of Inlife for specific subsamples in separate analyses. This was because we were unable to recruit the intended (n=122) number of caregivers. Finally, although participants were recruited on a national level, they may reflect a distinct subgroup from the general population. For instance, the online nature of the study inevitably resulted in a highly educated sample with a relatively high computer literacy and with highly motivated individuals who potentially already had a special interest in online tools [46,47]. This recruitment method that relied on self-selection probably introduced a selection bias, in which individuals who were more motivated and proficient regarding the use of digital tools than average were sampled, potentially resulting in more positive effects than would be found in a more representative sample. However, given the lack of positive results in this study, the potential impact of this selection bias appears to be minimal.

### Future Research Directions

First, future studies should determine methods to identify practical benefits, such as with qualitative research methods or momentary assessments in the flow of daily life. Efforts should be undertaken to develop research methods that can identify benefits in daily life that are ecologically valid for caregivers of people with dementia. Second, future studies should incorporate contextual factors, such as organizational implementation determinants and available social capital into the intervention design and implementation, to facilitate uptake and make use of the benefits of human interaction. It would be worthwhile to include other Inlife circle members and the people with dementia in the evaluation. Considering our promising qualitative findings in a caregiver subsample [38], we suggest adding more extensive qualitative research methods to gain more insight into the circumstances and factors that are required to make Inlife use effective. This might also enable tailoring the Inlife platform to individual needs by integrating persuasive design features, such as by providing personalized functionalities and tailored notifications that are relevant to the individual caregivers’ needs and are specific to their current stage in the caregiver process [48]. In this way, caregivers can become acquainted with the opportunities of the Inlife platform in accordance with their own needs and at their own pace.

### Conclusions

The present RCT demonstrated no significant effects of Inlife on feelings of caregiver competence, social support, and measures of caregiver well-being. Nevertheless, online tools such as Inlife show promise to facilitate the care process in daily life, though researchers should be modest regarding their effectiveness in terms of quantitative measures of well-being and quality of life. Future eHealth studies should (1) exploit the power of human interaction in eHealth and facilitate use of the user’s social capital, (2) apply extensive qualitative process evaluations to unravel beneficial effects for specific subgroups of caregivers and gain insight into potential barriers for implementation in clinical practice, and (3) from the start of the intervention’s development, carefully consider how interventions should be implemented by including contextual factors into the design and evaluation process. Applying these lessons can help researchers develop eHealth interventions for caregivers of people with dementia (such as Inlife) that are better suited to both carer needs and their wider implementation contexts.

## Appendix

Multimedia Appendix 1 CONSORT-eHEALTH checklist (V 1.6.1).

Multimedia Appendix 2 Sensitivity analyses of psychosocial outcome measures by group.

Multimedia Appendix 3 Change over time for feelings of competence (Short Sense of Competence Questionnaire [SSCQ]) for the intervention and control groups.

Multimedia Appendix 4 Change over time for perceived social support (Multidimensional Scale of Perceived Social Support [MSPSS]) for the intervention and control groups.

Multimedia Appendix 5 Baseline characteristics of the high active and low active Inlife users in the intervention group.

## Abbreviations

CarerQol: Care-Related Quality of Life Scale
HADS: Hospital Anxiety and Depression Scale
ICECAP-O: Investigating Choice Experiments for the Preferences of Older People Capability Measure for Older People
ITT: intention to treat
LMM: linear mixed model
LS: Loneliness Scale
LSNS-6: 6-item Lubben Social Network Scale
MSPSS: Multidimensional Scale of Perceived Social Support
PPT: per-protocol
PSS: Perceived Stress Scale
RCT: randomized controlled trial
SSCQ: Short Sense of Competence Questionnaire
SSL12-I: 12-item Social Support List-Interactions

## Data Availability Statement

The data that support the findings of this study are available on request from the corresponding author, HLC. The data are not publicly available due to restrictions (eg, their containing information that could compromise the privacy of research participants).
