# Supplementary material for: Lessons Learned From an Effectiveness Evaluation of Inlife, a Web-Based Social Support Intervention for Caregivers of People With Dementia: Randomized Controlled Trial
Source: JMIR Aging. 2022 Dec 7;5(4):e38656. doi: 10.2196/38656 (PMC9773030; doi:10.2196/38656)
Supplement: Multimedia Appendix 2 [file aging_v5i4e38656_app2.docx]

**Multimedia Appendix 2.** Sensitivity analyses^1^ of psychosocial outcome measures by group.

|  | **Control^3^**  **(N=46)** | **Inlife**  **High-active (N=23)** | B | SE | P | 95% CI | Effect size  Partial η2 | **Inlife**  **Low-active (N=20)** | B | SE | P | 95% CI | Effect size  Partial η2 |
| --- | --- | --- | --- | --- | --- | --- | --- | --- | --- | --- | --- | --- | --- |
|  | 16 wk. FU  Mean (SD) | 16 wk. FU  Mean (SD) |  |  |  |  |  | 16 wk. FU  Mean (SD) |  |  |  |  |  |
| **PPT** |  |  |  |  |  |  |  |  |  |  |  |  |  |
| SSCQ^#^ | 4.5 (1.7) | 3.7 (1.8) | -0.215 | 0.348 | 0.539 | -0.908 to 0.478 | 0.005 | 3.7 (2.1) | 0.117 | 0.364 | 0.750 | -0.608 to 0.841 | 0.001 |
| MSPSS^¶^ | 324.7 (152.1) | 306.1 (153.5) | -12.7 | 41.7 | 0.726 | -95.7 to 70.3 | 0.001 | 303.8 (175.8) | -18.9 | 42.6 | 0.659 | -103.6 to 65.8 | 0.002 |
| SSL-12 | 32.6 (7.5) | 29.6 (7.3) | -2.681 | 2.005 | 0.185 | -6.667 to 1.305 | 0.020 | 29.7 (8.2) | -2.800 | 2.016 | 0.168 | -6.808 to 1.207 | 0.022 |
| LS^^^ | 3.5 (3.9) | 4.5 (4.0) | 0.635 | 1.017 | 0.534 | -1.387 to 2.657 | 0.005 | 4.3 (3.8) | 0.649 | 1.039 | 0.534 | -1.416 to 2.714 | 0.005 |
| LSNS-6 | 18.4 (6.1) | 17.2 (6.1) | -1.277 | 1.680 | 0.450 | -4.618 to 2.065 | 0.007 | 17.0 (7.3) | -1.461 | 1.716 | 0.397 | -4.873 to 1.950 | 0.008 |
| PSS | 14.1 (8.0) | 15.2 (7.6) | 0.235 | 1.956 | 0.905 | -3.654 to 4.124 | 0.000 | 15.8 (6.1) | 1.347 | 1.997 | 0.502 | -2.624 to 5.318 | 0.005 |
| HADS | 22.9 (2.5) | 22.7 (3.0) | -0.355 | 0.683 | 0.604 | -1.712 to 1.002 | 0.003 | 22.2 (2.3) | -0.759 | 0.697 | 0.279 | -2.145 to 0.627 | 0.014 |
| PT^^^ | 4.5 (1.5) | 4.8 (1.1) | 0.355 | 0.366 | 0.335 | -0.373 to 1.082 | 0.011 | 4.3 (1.4) | -0.176 | 0.374 | 0.639 | -0.919 to 0.567 | 0.003 |
| CarerQol | 6.0 (2.1) | 5.9 (1.7) | -0.336 | 0.534 | 0.532 | -1.398 to 0.727 | 0.005 | 6.1 (2.3) | 0.045 | 0.546 | 0.934 | -1.040 to 1.130 | 0.000 |
| ICECAP-O^#^ | 0.8 (0.1) | 0.7 (0.2) | -0.009 | 0.025 | 0.719 | -0.059 to 0.041 | 0.002 | 0.8 (0.1) | -0.010 | 0.025 | 0.702 | -0.060 to 0,041 | 0.002 |

^1^The sensitivity analysis is based on a low active vs. high active user group split by the median of the total number of clicks on the Inlife platform (Median=590, Mean=1651, SD=2165, range 2-10699) [39].

^2^This table depicts linear regression analyses for the 16-week follow-up outcome measures adjusted for the age of the person with dementia .
^3^ The control group is the reference group, and 5 persons in the intervention group and 2 persons in the control group were lost to follow-up
^#^ adjusted for baseline scores, ^¶^ This variable was negatively skewed and a cubic transformation was used, ^^^ This variable was skewed, but no transformation could better approach a normal distribution. Therefore, raw data are presented, ^*^ P < 0.05. SSCQ Short Sense of Competence Questionnaire, MSPSS Multidimensional Scale of Perceived Social Support, SSL-12 Social Support List 12-Interactions, LS Loneliness Scale, LSNS-6 Lubben Social Network Scale, HADS Hospital Anxiety and Depression Scale, ICECAP-O Investigating Choice Experiments for the Preferences of Older People Capability measure for Older People, CarerQol Care and Quality of Life scale, IRS: custom scale interpersonal relationships PSS Perceived Stress Scale, PT perseverance time
