# Supplementary figures and images for: Lessons Learned From an Effectiveness Evaluation of Inlife, a Web-Based Social Support Intervention for Caregivers of People With Dementia: Randomized Controlled Trial
Source: JMIR Aging. 2022 Dec 7;5(4):e38656. doi: 10.2196/38656 (PMC9773030; doi:10.2196/38656)

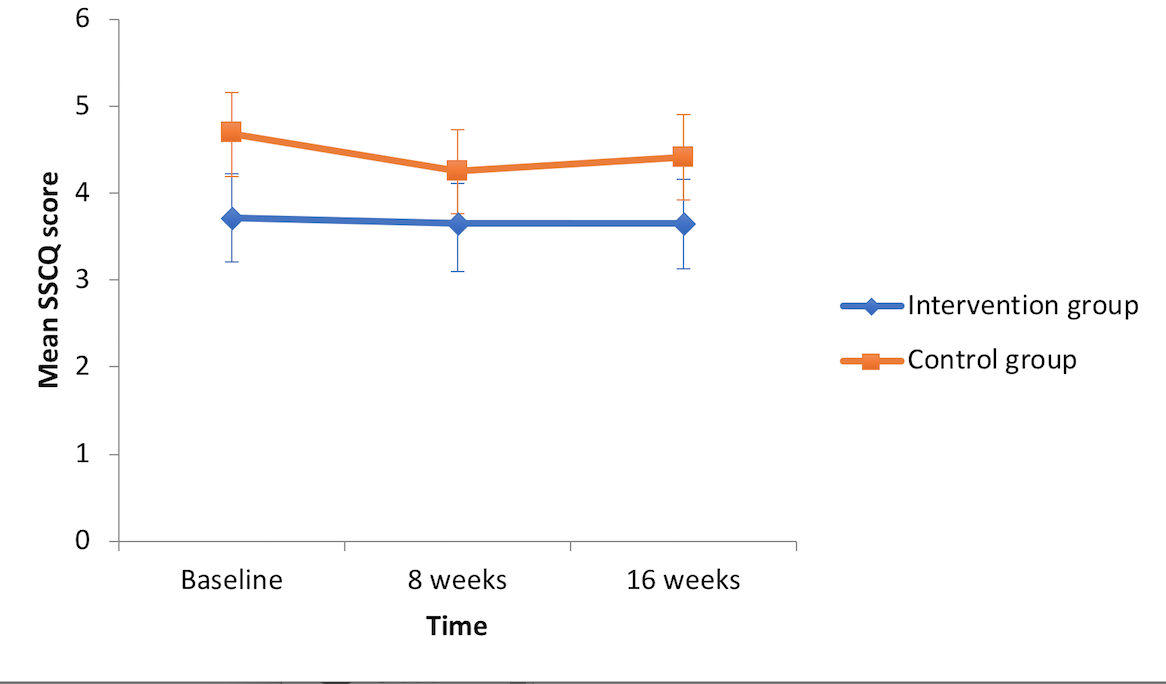

Supplement: Multimedia Appendix 3 [file aging_v5i4e38656_app3.png]

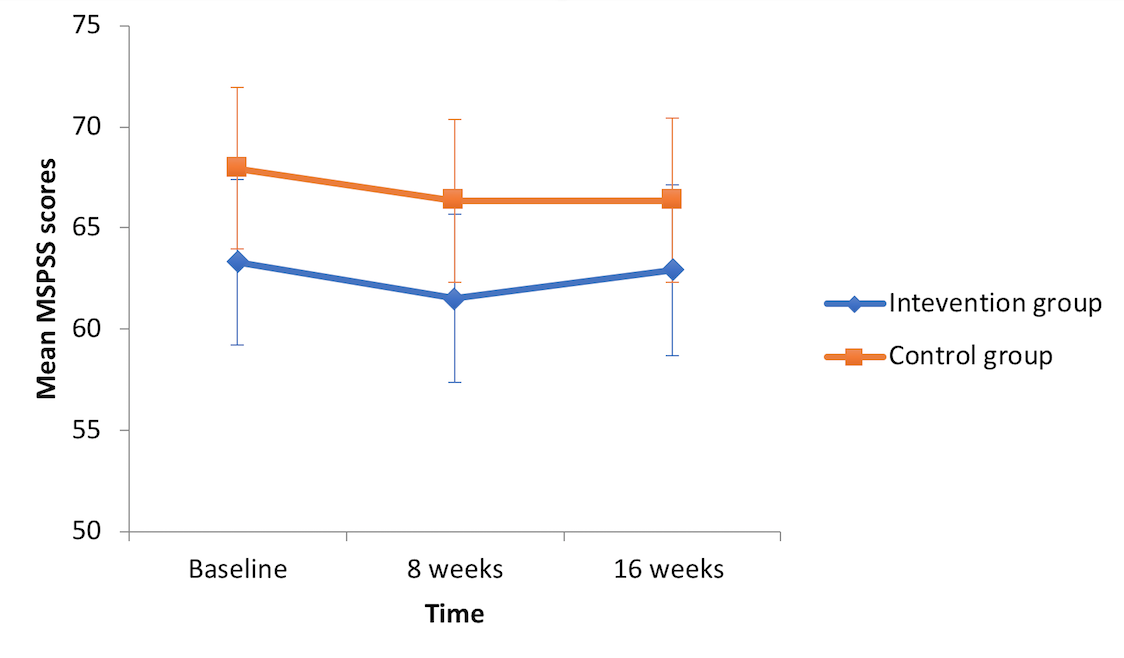

Supplement: Multimedia Appendix 4 [file aging_v5i4e38656_app4.png]
