# Supplementary material for: Lessons Learned From an Effectiveness Evaluation of Inlife, a Web-Based Social Support Intervention for Caregivers of People With Dementia: Randomized Controlled Trial
Source: JMIR Aging. 2022 Dec 7;5(4):e38656. doi: 10.2196/38656 (PMC9773030; doi:10.2196/38656)
Supplement: Multimedia Appendix 5 [file aging_v5i4e38656_app5.docx]

**Multimedia Appendix 5.** Baseline characteristics of the high active and low active Inlife users in the intervention group^1^.

| **Variable** | **High-users (n=23)**  Mean (SD) | **Low-users (n=25)**  Mean (SD) | P |
| --- | --- | --- | --- |
| Age (range) | 58.0 (11.7, 26-84) | 58.2 (12.2, 31-78) | 0.973 |
| Sex (n, %) |  |  | 0.263 |
| Male | 10 (43.5) | 7 (28.0) |  |
| Female | 13 (56.5) | 18 (72.0) |  |
| Years of education | 13.2 (5.0) | 12.7 (5.2) | 0.519 |
| Years of caring | 7.5 (10.3) | 5,0 (4.3) | 0.274 |
| Hours of caring per week | 34.6 (42.6) | 33.3 (47.9) | 0.925 |
| Caregiver relationship (n, %) |  |  | 0.244 |
| Spouse/partner | 9 (39.1) | 12 (48.0) |  |
| Son/Daughter (-in law) | 11 (47.8) | 13 (52.0) |  |
| Brother/ sister | 1 (4.4) | - |  |
| Other^a^ | 2 (8.7) | - |  |
| Circle members (n, %) | 9.4 (5.2) | 3.3 (3.7) | 0.000*** |
| Age of care recipient (SD. range) | 73.7 (13.0, 47-90) | 76.2 (8.4, 56-91) | 0.437 |
| Years of education of care recipient | 10.2 (4.8) | 10.1 (5.0) | 0.946 |
| Type of Dementia (n, %) |  |  | 0.336 |
| Alzheimer’s disease | 11 (47.8) | 14 (56.0) |  |
| Frontotemporal dementia | 3 (13.1) | 2 (8.0) |  |
| Vascular Dementia | 4 (17.4) | 2 (8.0) |  |
| Dementia with Lewy bodies | - | 1 (4.0) |  |
| Mixed dementia | 1 (4.3) | 1 (4.0) |  |
| Dementia NAO | 4 (17.4) | 5 (20.0) |  |
| Living situation (n, %) |  |  | 0.178 |
| Home | 18 (78.3) | 23 (92.0) |  |
| Nursing home | 5 (21.7) | 2 (8.0) |  |
| **Outcome variables at baseline** |  |  |  |
| SSCQ | 4.0 (2.0) | 3.6 (1.8) | 0.505 |
| MSPSS | 65.7 (11.0) | 61.1 (14.5) | 0.224 |
| SSL-12 | 31.8 (6.6) | 28.0 (7.2) | 0.060 |
| LS | 3.8 (3.8) | 4.0 (3.2) | 0.895 |
| LSNS-6 | 17.0 (6.6) | 16.7 (6.6) | 0.965 |
| PSS | 14.3 (7.1) | 14.1 (6.2) | 0.928 |
| HADS | 22.7 (2.1) | 23.0 (2.3) | 0.636 |
| PT | 5.4 (1.0) | 5.4 (0.8) | 0.918 |
| CarerQol | 10.0 (2.4) | 9.4 (2.5) | 0.899 |
| ICECAP-O | 0.8 (0.2) | 0.8 (0.1) | 0.731 |
| ^1^ The low active vs. high active user groups are based on a median split of the total number of clicks on the Inlife platform (Median=590, Mean=1651, SD=2165, range 2-10699) [38] * P < 0.05, ***P < 0.001 SSCQ Short Sense of Competence Questionnaire, MSPSS Multidimensional Scale of Perceived Support, SSL-12 Social Support List 12-Interactions, LS Loneliness Scale, LSNS-6 Lubben Social Network Scale, HADS Hospital Anxiety and Depression Scale, ICECAP-O Investigating Choice Experiments for the Preferences of Older People Capability measure for Older People, CarerQol Care and Quality of Life scale, PSS Perceived Stress Scale, PT perseverance times | | | |
